# Mental Health of Children With Attention Deficit and Hyperactivity Disorder and Their Parents During the COVID-19 Lockdown: A National Cross-Sectional Study

**DOI:** 10.3389/fpsyt.2022.902245

**Published:** 2022-06-13

**Authors:** E. Bobo, E. Fongaro, L. Lin, C. Gétin, L. Gamon, M-C. Picot, D. Purper-Ouakil

**Affiliations:** ^1^CHU Montpellier, Saint Eloi Hospital, Unit for Child and Adolescent Psychiatry (MPEA1), Montpellier, France; ^2^Center for Research in Epidemiology and Population Health (CESP), INSERM U 1018, Paris-Saclay University, Villejuif, France; ^3^Clinical Research and Epidemiology Unit, Department of Medical Information, University Hospital Medical Center, Montpellier, France; ^4^Hyper-Super, TDAH France, Paris, France

**Keywords:** COVID-19, Attention Deficit Hyperactivity Disorder (ADHD), child, lockdown, depressive disorder, parental coping strategies, behavioral symptoms

## Abstract

The coronavirus disease 2019 (COVID-19) pandemic has caused a real disruption of children's lives. Children with neurodevelopmental disorders and their parents seem to be particularly vulnerable to adverse mental health effects due to lockdown policies. This study explores the psychological state of children with Attention Deficit Hyperactivity Disorder (ADHD) and their parents during the first lockdown in France. A national prospective cross-sectional parent-reported study was conducted using an online survey disseminated through different social networks of French ADHD associations during the first lockdown. The survey consisted of open-ended, multiple-choice questions and standardized questionnaires such as the Strengths and Difficulties Questionnaire (SDQ), the coping self-report questionnaire (Brief COPE) and the Patient Health Questionnaire-2 (PHQ-2). A total of 538 parents completed the online survey between the 6th and the 15th of April 2020. These results suggest that most children (65.29%) did not experience a worsening of their behavior but still had pathological levels of hyperactivity (56.47%) and behavioral (57.60%) symptoms at the time of the first lockdown. In addition, some parents (26.27%) showed responses indicating possible major depressive disorder. Positive parental coping strategies were associated with both improved child behavior and fewer parental depressive symptoms. Strengthening parents' coping strategies may be an effective intervention to protect both parents and children with ADHD from the negative psychological effects of lockdown. In times of pandemic, psychological care modalities must evolve to provide quality online interventions for families of children with ADHD.

## Introduction

The COVID-19 global health emergency has impacted people around the world in many aspects. While adults have been more likely to suffer the most severe health consequences, children and adolescents were affected in different ways by the pandemic. The strict national and regional containment measures implemented in many countries imposed school and activities closures, requiring students to stay at home. In the general population, with the progression of the pandemic, negative changes of the children's mental health have been documented, especially as regards increased anxiety and depressive symptoms (1–5). Several factors, such as gender, social isolation, difficult parent-child relationships, socioeconomic disadvantage, and increased media information seeking about COVID have been related to negative emotions (6).

Among children and adolescents, those with Attention Deficit Hyperactivity Disorder (ADHD) are potentially a vulnerable group to the effects of social isolation. In the context of the pandemic, the abrupt cessation of schooling, the increase in family time imposed by the containment measures and the potential anxiety-provoking nature of this health and economic crisis are all factors that may influence the symptomatology of children and adolescents with ADHD (7). A recent review showed a moderate psychological impact of lockdown on children with neurodevelopmental disorders but a greater psychological distress in parents, especially when children presented with an autism spectrum disorder (8).

We conducted a national cross-sectional survey among parents of ADHD children during the strictest French lockdown implemented from March 16 to May 12, 2020. The results about parents' perceptions of their child's mental health and relationship to care during this period of confinement have been previously reported (9).

The aim of the present study was to examine the psychological impact of the COVID-19 lockdown on children with ADHD and their parents to understand this population's needs and provide appropriate support. We hypothesized that ADHD symptoms and worsening of the child's global state would be related to depressive symptoms in the parent while active coping and better environmental conditions would be negatively associated with parental depressive symptoms.

## Materials and Methods

### Study Design and Population

A national cross-sectional study was conducted at the time of the complete lockdown in France. Parents of children with ADHD were invited to fill out an online survey posted on social networks of ADHD family support associations. A total of 538 parents responded to the online survey between the 6th and the 15th of April 2020.

### Procedures

An online survey was created using the Formstack platform and distributed via social media by French ADHD associations. The survey included information to parents about the study objectives and the use of collected data. Data collection began on the twentieth day of lockdown and lasted 9 days.

### Measures

The survey included self-report questionnaires, closed-ended and open-ended questions. The Patient Health Questionnaire-2 (PHQ-2) and the BRIEF COPE are standardized self-report questionnaires that assess, respectively, parents' depressive symptoms and the different coping strategies used by parents to deal with the imposed lockdown and pandemic (10, 11). The PHQ2 inquiries about the frequency of depressed mood and anhedonia over the past 2 weeks. Each item ranges from 0 (“not at all”) to 3 (“nearly every day”). A PHQ-2 score greater than or equal to 3 detects major depression with a sensibility of 83% and a specificity of 92%, (10). The BRIEF COPE self-questionnaire allowed us to evaluate the different coping strategies the parent used to deal with this initial confinement situation. This instrument proposes 14 scales evaluating distinct dimensions of coping. In order to reduce the time required to complete the test, we selected the 8 coping scales correlated with the perception of control or evolution (favorable or unfavorable) of the stress situation. The scales selected were active coping, denial, emotional support seeking, behavioral disengagement, humor, acceptance, blame and expression of feelings. Each item ranges from 1 to 4, according to the frequency of the parental coping strategy used (11). The emotional, behavioral, and cognitive aspects of the ADHD child were assessed using the Strengths and Difficulties Questionnaire (SDQ). The SDQ is one of the most widely used questionnaires to identify psycho-pathological problems in children and adolescents. We used the SDQ questionnaire to assess the difficulties of the ADHD child during confinement on the emotional (SDQ-E), conduct (SDQ-C) and hyperactivity (SDQ-H) dimensions (12). This instrument is composed of 5 scales assessing the child's psychopathological difficulties during the last two weeks. The social dimension could not be assessed due to the confinement. Each dimension has a difficulty score between 0 and 10. Behavioral, emotional, or hyperactivity difficulties were designated as pathological when the scores were strictly > 3, 4, or 6, respectively. A total difficulty score was calculated excluding the social dimension. In our study, a total SDQ strictly > 13 was indicative of psychopathological difficulties in the child or adolescent concerned. The survey's closed questions collected the demographic characteristics of the participants as well as their family, social and financial living conditions during this specific period of lockdown. Parents were also asked about the evolution of their child's medical and psychological follow-up, the evolution of their child's behavior, the use of medication for ADHD, and the presence of reactive stress symptoms in their child (physical manifestation of stress, sleep disturbances, irritability, etc.).

For example, the question “How has your child's behavior changed since the containment measures?” asked parents to provide information on changes in their child's behavior by selecting one of the following three items: “Worsening of my child's behavior,” “No change in my child's behavior” or “Improvement in my child's behavior.”

### Statistical Analysis

Continuous parametric data are presented as means ± standard deviation (SD), and categorical variables as numbers and percentages. To identify factors related to depressive symptoms in parents, a logistic regression was used. Factors selected from the univariate analyses (*p* < 0.20) were entered into the multivariate model and selected using a backward selection method using the maximum likelihood ratio test. An adjusted odds-ratio (OR) and its 95% confidence intervals were reported. The same method was used to identify the risk factors of a worsening of the child's behavior. Statistical analyses were performed with SAS software version 9.1 (SAS Institute, Cary, NC, USA).

### Ethics

The survey being completely anonymous, no ethics committee was involved. The respondents were informed about the objectives of the survey when opening the link, before accessing the questionnaire.

## Results

### Socio-Demographic and Contextual Data

Families who responded for more than one child at a time (*n* = 5), or patients who responded for themselves (*n* = 2) were excluded from the analysis to allow comparability of parents' responses. A total of 533 families were included in the final analysis. Most respondents were female (95 % CI: 93.5; 97.2) and had male children (86.7 %) with a mean age of 10.5 years (95% CI: 7.6; 13.4). Most parents were well informed about the health situation and containment rules 89.9% and had a trusted person who could come to their aid if needed (61.3%). Twenty-four percentage of the population had a relative who had been affected by the coronavirus. All sociodemographic and contextual data are summarized in Table 1.

**Table 1:** Description of the population.

**General characteristics**	**Frequency *N*= 533**	**Percent %**
**Sex of parent responding to questionnaire**		
Woman	510	95.7
Man	23	4.3
**Sex of child**		
Girl	58	13.3
Boy	377	86.7
**Child's Age**		
Mean (+- SD)	513	10.5 (2.9)
**Environmental conditions of containment**		
The parent lives alone with the child	101	18.9
The parent feels cramped in their home	103	19.3
The parent has a support person who can come and help if needed	327	61.3
Parent has an occupation at risk for COVID-19 contamination	121	22.7
Parent is afraid of lack of provisions/ financial resources	113	21.2
Parent is well informed about the health situation and the containment rules	479	89.9
A parent or relative has been affected by the coronavirus	128	24.0

### Psychological Condition of Children: Description and Associated Risks

Among the 533 participants, most parents observed changes in their children's emotional state and behaviors during the quarantine. One-third of parents reported worsening of their child's behavior (34.7%), no visible change in behavior (34.3%) and improvement in behavior (31.0%), respectively. More than half of the children had hyperactivity (56.5%) and behavior (57.6%) scores above the clinical cutoff. One third of the children had a pathological emotional score (32.3%). Half of children were on medication for their ADHD (54.6%) and were not requiring immediate medical or psychological follow-up (57.7%). Most children were concerned about the pandemic (55.0%) and worried about the health of their loved ones (52.2%). Many parents reported sleep problems (54.6%) and irritability (50.1%) in their children, and 64.5% reported poor compliance with public health measures (Table 2).

**Table 2:** Description of the psychological state of the children.

				**Evolution of the child's behavior**							
				**Worsening of** **behavior**		**No visible** **changes**		**Improvement** **of his behavior**			
		**Total population**		***n***	**%**	***n***	**%**	***n***	**%**		
**Total population**		***N*** **=** **533**	**%**	**185**	**34.7**	**183**	**34.3**	**165**	**31.0**	**Test**	***p***
**Child's characteristics**											
SDQ^*^	Emotional	172	32.3	96	51.9	41	22.4	35	21.2	CHI2	<0.01
	Behavioral	307	57.6	159	85.9	96	52.5	52	31.5	CHI2	<0.01
	Hyperactivity	301	56.5	142	76.8	97	53.0	62	37.6	CHI2	<0.01
	Total score	320	60.0	164	88.6	97	53.0	59	35.8	CHI2	<0.01
**ADHD Follow-up**	Did not need	307	57.7	77	41.6	119	65.0	111	67.7	CHI2	<0.01
	Did not seek	64	12.0	21	11.4	24	13.1	19	11.6		
	Has deteriorated	161	30.3	87	47.0	40	21.9	34	20.7		
**ADHD Medication**	Taking ADHD Medication	291	54.6	94	50.8	107	58.5	90	54.5	CHI2	0.34
**Parent's characteristics**											
**Coping Strategy**	Active coping	412	77.3	137	74.1	136	74.3	139	84.2	CHI2	0.04
	Acceptance	500	93.8	164	88.7	174	95.1	162	98.2	CHI2	<0.01
	Emotional support	185	34.7	70	37.8	49	26.8	66	40.0	CHI2	0.02
	Venting	219	41.1	70	37.8	67	36.6	82	49.7	CHI2	0.03
	Denial	51	9.6	23	12.4	14	7.7	14	8.5	CHI2	0.25
	Self-blame	39	7.3	20	10.8	10	5.5	9	5.5	CHI2	0.08
	Humor	115	21.6	34	18.4	27	14.8	54	32.7	CHI2	<0.01
	Behavioral disengagement	78	14.6	32	17.3	26	14.2	20	12.1	CHI2	0.38
PHQ-2^**^	Depressive Symptoms	140	26.3	74	40.0	38	20.8	28	17.0	CHI2	<0.01

* *SDQ Strengths and Difficulties Questionnaire*.

#x0002A;*: *PHQ-2 Depressive Symptoms*.

Univariate analysis showed no link between taking ADHD medication and changes in child behavior (p=0.34) but did show a strong relationship between worsening medical or psychological follow-up and worsening child behavior (47.0% vs. 21.9 vs. 20.7%). These results are visible on Table 2.

Multivariate logistic regression significantly showed that deterioration of the medical or psychological follow-up (AOR, 2.63; 95%CI, 1.58–4.37), an overall pathological psychological state in the child (AOR, 5.51; 95%CI, 3.14–9.65) and parental depression (AOR, 2.00; 95%CI, 1.20–3.33) as risk enhancing correlates for worsening of the child's behavior during lockdown. However, parents who frequently used humor as a coping strategy were more likely to see improvement in their child's behavior (AOR, 2.51; 95%CI, 1.47–4.30), (Table 3).

**Table 3:** Factors associated with change in the child's behavior.

		**Worsening of behavior**			**Improvement of behavior**			***p*-value**
		**OR**	**95% CI**	***p*-value**	**OR**	**95% CI**	***p*-value**	
ADHD Follow-up	Did not need	1	-		1	-		0.0007
	Did not seek care	0.87	[0.43–1.76]	0.07	1.12	[0.56–2.23]	0.87	
	Has deteriorated	2.63	[1.58–4.37]	0.0001	1.11	[0.64–1.93]	0.87	
SDQ total score	Non-pathological	1	-		1	-		<0.0001
	pathological	5.51	[3.14–9.65]	<0.0001	0.52	[0.33–0.82]	0.005	
The parent has a support person	No	1	-		1	-		0.044
	Yes	0.66	[0.41–1.06]	0.09	1.28	[0.79–2.08]	0.32	
PHQ-2^*^	No	1	-		1	-		0.008
	Yes	2.00	[1.20–3.33]	0.008	0.92	[0.52–1.63]	0.78	
Emotional support coping	Not used	1	-		1	-		0.053
	used	1.60	[0.98–2.63]	0.06	1.70	[1.06–2.74]	0.03	
humor coping	Not used	1	-		1	-		0.003
	Used	1.71	[0.93–3.15]	0.08	2.51	[1.47–4.30]	0.001	

*Reference modality: No change in behavior*.

* *PHQ-2 Depressive Symptoms*.

### Psychological Condition of Parents: Description and Associated Risks

Nearly one third of parents (26.3%) had a significantly elevated PHQ-2, indicating presence of depressive symptoms. Most parents used active (77.3%) and acceptance coping strategies (93.8%). A smaller proportion of parents used instrumental support (34.7%), humor (21.6%), venting (41.1%), denial (9.6%), self-blame (7.3%), or behavioral disengagement (14.6%) as coping strategies (Table 2).

In multivariate analysis, parents using active coping strategies had less risk of showing depressive symptoms (AOR = 0.52; 95%CI, 0.32–0.85). In contrast, parents feeling cramped at home (AOR = 2.64; 95%CI, 1.59–4.40), being concerned about any shortage of supply or financial resources (AOR =3.44; 95%CI, 2.10–5.61) or often using denial coping strategies (AOR = 2.43; 95%CI, 1.23–4.77) had a higher risk of depressive symptoms. Moreover, an overall pathological psychological state in the child (AOR = 2.59; 95%CI, 1.60–4.21) and having an older child (AOR = 1.13; 95%CI, 1.04–1.21) were significantly associated with a higher risk of depressive symptoms in the parent (Table 4).

**Table 4:** Factors associated with parental depressive symptomatology.

		**Total**	**Major Depressive Episode**			
		***N* = 533 (%)**	***N* = 140 (26.3%)**	**OR**	**95% CI**	***P*-Value**
Child Age	Mean (SD)	10.5 (2.9)	10.9 (3.14)	1.13	[1.04–1.21]	0.0020
SDQ total score^*^	Non-pathological (%)	213 (40.0)	31 (22.1)	1	[Reference]	0.0001
	Pathological (%)	164 (60.0)	109 (77.9)	2.59	[1.60–4.21]	
Feeling crowded in their home	No (%)	430 (80.7)	92 (65.7)	1	[Reference]	0.0002
	Yes (%)	103 (19.3)	48 (34.3)	2.64	[1.59–4.40]	
Fear of lack of provisions/ resources	No (%)	113 (21.2)	83 (59.3)	1	[Reference]	<0.0001
	Yes (%)	320 (78.8)	57 (40.7)	3.44	[2.10–5.61]	
Uses active coping	No (%)	121 (22.7)	46 (32.9)	1	[Reference]	0.0087
	Yes (%)	412 (77.3)	94 (67.1)	0.52	[0.32–0.85]	
Uses coping denial	No (%)	482 (90.5)	116 (82.9)	1	[Reference]	0.0101
	Yes (%)	51 (9.6)	24 (17.1)	2.43	[1.23–4.77]	

*Reference modality: No Major Depressive Episode*.

* *SDQ Strengths and Difficulties Questionnaire*.

## Discussion

### Results in Perspective

This study set out to explore how behavior of children with ADHD changed within the first national lockdown due to COVID-19 pandemic in France. Notably, the results showed that two-third of children had improved or unchanged behavior according to their parents. This finding is consistent with results by Waite and al. that showed that some groups of children reported mental health benefits during lockdown (2). Specifically, this population-based study conducted in the UK showed that behavioral and hyperactivity difficulties scores significantly decreased in children with neurodevelopmental disorders compared to those without. It was not clear if these results were mainly due to school closures and homeschooling, increased parental presence, increased play time or social disruption. Nevertheless, the qualitative analysis of our survey previously published, revealed that parents related their child's improvement to the reduction of on-site school time, which is a known source of social stress and learning difficulties for children with ADHD (9). Accordingly, the lockdown condition may have eased certain negative emotional consequences of ADHD.

On the other side, as expected, half of children had elevated mental health symptoms during the lockdown, with significant scores of behavioral problems and hyperactivity/inattention. In fact, prior mental health is one of the most prominent predictors of child mental health difficulties (7). A recent study from Canada suggested that children who struggled with mental health symptoms prior to COVID-19 were also struggling during the pandemic (6). Furthermore, a meta-analysis showed high levels of anxiety and depression in children during the COVID-19 pandemic, exacerbated by older age of the child and the duration of the pandemic (3).

Our survey also explored which indicators were associated with parental depression and a change in child behavior respectively.

Poor medical-psychological follow-up was associated with worsening of children's behavior. Accordingly, families who received remote medical attention related a good experience with it (9, 13). These results are in line with different studies showing effectiveness and satisfaction with telehealth for adults and children with ADHD and other conditions highlighting the need for remote medical care when usual care is not available (14–16).

This survey showed no link between taking ADHD medications and changes in child's behavior during lockdown. As we had no information whether ADHD medication was recently implemented, and due to the cross-sectional nature of the date, this finding should be interpreted with caution. On the other hand, parents who usually involved humor or emotional support as coping strategies, reported less deterioration of their child's behavior. Therefore, we assume that parents with good coping strategies were probably more likely to create structured daily schedules and mechanisms of positive reinforcement tailored to their child's specific needs. Programs targeting parental coping strategies in times of crisis may be useful both for parents and their children with ADHD.

Most children with ADHD did not experience a worsening of their behavior during the first COVID-19 lockdown; nevertheless, they still maintained elevated behavioral symptoms. In addition, a significant proportion of parents exhibited depressive symptoms. The results showed that a pathological level of symptoms or older age in children were associated with a higher risk of parental depression during lockdown. These findings are consistent with a recent study that highlighted the association between internalizing and externalizing symptoms in children with parental stress, depressive states, and anxiety in parents during lockdown (17, 18). In this study, the parents with active coping strategies were less likely to develop depressive episodes during lockdown. This is in line with the benefits of interventions designed to facilitate the adjustment of parents of children with ADHD in improving child's behavioral problems (19). **C**hildren's maladaptive behaviors and low parental resilience are two factors that can affect the family's quality of life (17, 20). Thus, it seems essential to act simultaneously on the child's symptoms and the parents' coping strategies to improve the quality of life of families having children or adolescents with ADHD.

### Study Limitations

Our findings must be interpreted considering the study design limitations, which did not assess how behaviors of children with ADHD changed in relation to those of the general population. It is also important to highlight that the study population was not a representative sample. We can assume there was a bias toward families involved in ADHD associations, and thus better engaged and supported in their parenting practices. For this reason, we expect the levels of parental depression reported here to be an underestimate of the extent of depression experienced during lockdown. Another limitation to this study is inherent in the parental assessment of the child's behaviors and manifestation of ADHD symptoms. The increase in family time during the lockdown may have altered parental perception of the child. This phenomenon may have played a role on the evaluation of the child's symptoms. The present study took place at the early stage of the first lockdown. Thus, the results of this study reflected the first month of lockdown and cannot claim to evaluate the effects of long-term lockdown and other pandemic-related stresses. Furthermore, people without internet access and non-French speakers are most likely not represented in this study.

### Study Implications

Even though most children with ADHD did not show a deterioration of their mental state during the first lockdown, their levels of hyperactivity and behavioral symptoms were significant. Strengthening parents' coping strategies could be an effective intervention to protect the nuclear family from the negative psychological effects of the lockdown. Several resources have been provided to parents and professionals in order to meet this objective (21). In times of pandemic, medical psychological care modalities must evolve to provide

## Data Availability Statement

The original contributions presented in the study are included in the article, further inquiries can be directed to the corresponding author.

## Ethics Statement

Ethical review and approval was not required for the study on human participants in accordance with the local legislation and institutional requirements. The patients/participants provided their written informed consent to participate in this study.

## Author Contributions

All authors contributed to the design and conceptualization of the study, which was coordinated by DP-O. EB, EF, and DP-O contributed to the literature review. M-CP, LL, and LG contributed to the data analyses and formulation of the manuscript, with input from all other authors. EB drafted the manuscript. EF critically revised the manuscript and DP-O revised and approved the final version of the manuscript. All authors contributed to the article and approved the submitted version.

## Conflict of Interest

During the last 3 years, DP-O reports non-financial support from Medice and Shire, nonfinancial support from HAC Pharma, outside the submitted work. The remaining authors declare that the research was conducted in the absence of any commercial or financial relationships that could be construed as a potential conflict of interest.

## Publisher's Note

All claims expressed in this article are solely those of the authors and do not necessarily represent those of their affiliated organizations, or those of the publisher, the editors and the reviewers. Any product that may be evaluated in this article, or claim that may be made by its manufacturer, is not guaranteed or endorsed by the publisher.

## Abbreviations

ADHD: Attention Deficit Hyperactivity Disorder.
